# Lipoprotein(a) at a “Tipping Point”: case to move to universal screening

**DOI:** 10.1016/j.ajpc.2025.101274

**Published:** 2025-08-22

**Authors:** Harpreet S. Bhatia

**Affiliations:** Division of Cardiovascular Medicine, University of California San Diego, La Jolla, CA, USA

**Keywords:** Lipoprotein(a), Lipids, Prevention

## Abstract

Elevated lipoprotein(a) [Lp(a)] is well established as a common risk factor for atherosclerotic cardiovascular disease (ASCVD). Lp(a) levels are >90 % genetically determined. However, Lp(a) remains very underrecognized as a cardiovascular risk factor with low rates of testing. In this article, the case for universal Lp(a) screening is outlined including the high yield of a single test and the relative stability in levels and risk categories over time resulting in a need to test most people once. Additionally, Lp(a) testing impacts clinical management. At a minimum, elevated Lp(a) is associated with multiple cardiovascular diseases and Lp(a) measurement may be incorporated into more precise individual risk assessment. Elevated Lp(a) should prompt more aggressive risk factor modification, particularly low-density lipoprotein-cholesterol (LDL-C) lowering and a preference for Proprotein convertase subtilisin/kexin type 9 inhibitors (PCSK9i). There may also be a role for aspirin use in primary prevention based on currently available evidence. Identification of elevated Lp(a) also enables cascade screening to further identify affected individuals and helps to enable further research and individuals who may be candidates for novel therapies. While there are strategies to address increased cardiovascular risk in individuals with elevated Lp(a) today, it is clear that residual risk remains, and there are several novel, targeted therapies for lowering Lp(a) that are in advanced stages of development, which are also reviewed. Lp(a) remains underappreciated and undertested in clinical practice, and there are several arguments in favor of testing today with hope for potent targeted therapies for Lp(a)-lowering in the very near future.

## 1 Introduction

Lipoprotein(a) [Lp(a)] is well established as a genetic risk factor for atherosclerotic cardiovascular disease (ASCVD) and calcific aortic valve disease when present at elevated levels. Elevated Lp(a) is common, with an estimated prevalence of levels >50 mg/dL or 125 nmol/L in 20 % of the global population [1]. However, international guidelines vary in their recommendations regarding Lp(a) testing. The 2018 Multi-Society Cholesterol Guidelines make mention of Lp(a) as a risk enhancer, but do not provide a statement regarding universal screening [2]. The more recent European Society of Cardiology (ESC) / European Atherosclerosis Society (EAS) 2019 guidelines recommend measurement of Lp(a) once in every adult [3]. The Canadian Cardiovascular Society (CCS) guidelines also recommend testing in all adults [4]. Most recently, the National Lipid Association (NLA) scientific statement on Lp(a) recommended testing at least once in all adults [5], the first major US society to do so.

However, Lp(a) remains very underrecognized as a cardiovascular risk factor. Several studies have examined rates of Lp(a) testing across different regions and populations, with multiple observing rates of testing of <1 % of all adults [6]. In addition to discrepancies in guidelines, one of the likely barriers to more widespread testing for Lp(a) is uncertainty regarding the management of individuals with elevated Lp(a), or the notion that it does not affect clinical management because targeted therapies are not yet available.

In this review, the case for universal Lp(a) screening will be outlined (Central Illustration), including the utility of a single test, the use of Lp(a) in risk assessment, and strategies for management of individuals with elevated Lp(a) today. An overview of the future of Lp(a) management involving novel, targeted therapies in advanced stages of development will also be discussed.

**Central Illustration:**
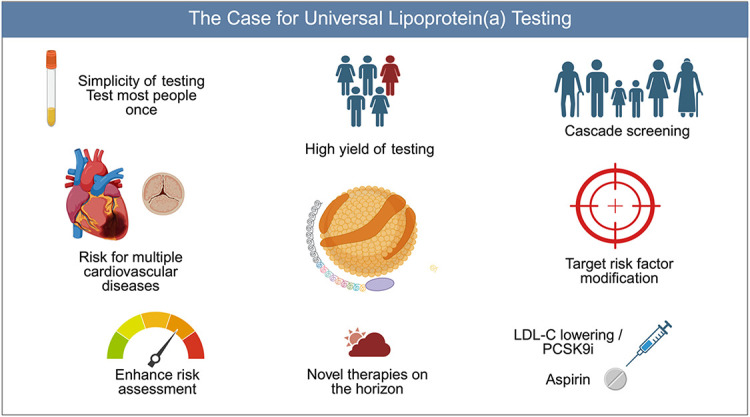
Arguments in favor of universal lipoprotein(a) testing are outlined including the simplicity and high yield of testing and need to test most people once, identification of risk for multiple cardiovascular diseases, enhancing individualized risk assessment and impact on clinical management including targeting aggressive risk factor modification including LDL-C lowering with use of PCSK9i, aspirin therapy, and enabling cascade screening. Finally, there are several novel, potent, targeted therapies in advanced stages of development.

## 2 The case for universal screening

There are several reasons why Lp(a) should be tested in all adults at least once. To start with, elevated Lp(a) is very common, depending on the definition. A level of >50 mg/dL or >125 nmol/L is considered high risk, and this was primarily based on a European population-based study where this represented the top 20 % of individuals. In a large study of >500,000 individuals referred for Lp(a) testing in the United States, Lp(a) >50 mg/dL was present in 24 % of individuals [7]. Thus, there is a high yield to Lp(a) testing for identifying individuals at increased risk.

Lp(a) levels are >90 % genetically determined by the *LPA* gene [8] and there is a limited impact of lifestyle on Lp(a) levels [9], making Lp(a) measurement simple and effective for screening. While it is increasingly recognized that Lp(a) levels vary over time and that a small group of conditions may impact Lp(a) levels, Lp(a) levels generally remain quite stable over time. In a study of >16,000 individuals from the UK Biobank with serial Lp(a) testing, there was a very strong correlation between Lp(a) levels over a median of 4.4 years, and the change in levels over time was not independently predictive of coronary artery disease risk [10]. Additionally, the change in Lp(a) levels over time may be most relevant in individuals with borderline levels. In a study of nearly 12,000 individuals from the Mayo Clinic, individuals were categorized by Lp(a) level into normal (<30 mg/dL), borderline (30–50 mg/dL) and high (≥50 mg/dL) Lp(a). Over 4.5 years of follow-up, 96 % of those with normal Lp(a) (63 % of participants) and 90 % of those with high Lp(a) (26 % of participants) remained in these categories, while 51 % of those with borderline Lp(a) (11 % of participants) changed categories in follow-up. Thus, 89 % of participants remained in the same Lp(a) category over time [11]. In a long-term follow-up study of participants in the Women’s Health Study, a single Lp(a) measure was predictive of major adverse cardiovascular event (MACE) risk 30 years later [12]. Together, these findings emphasize the stability and utility of a single Lp(a) measurement.

The question often arises of how Lp(a) measurement changes management. At the minimum, Lp(a) is associated with multiple cardiovascular diseases including coronary disease, aortic valve disease, heart failure, stroke and peripheral arterial disease [13]. Importantly, Lp(a) is associated with risk in a continuous manner, independent of low-density lipoprotein-cholesterol (LDL-C) [14]. It is also associated with increased risk regardless of the number of modifiable risk factors [15], and even in otherwise low risk individuals [16].

Additionally, Lp(a) measurement may be incorporated into a more precise risk assessment at the individual level in the primary prevention setting. Every 50 nmol/L increment of Lp(a) (or approximately 20 mg/dL) is associated with an 11 % increase in ASCVD risk, independent of traditional risk factors [17]. A framework for incorporating this into risk assessment has been proposed in the AHA scientific statement about Lp(a) by multiplying an individual’s traditional 10-year risk estimate by 1.11^(Lp(a) level in nmol/L / 50)^ [18]. It has recently been shown that Lp(a) is associated with risk independent of the new AHA PREVENT equations; though the addition of Lp(a) does not significantly improve risk prediction on top of these equations at the population level, Lp(a) may be used to refine risk assessment at the individual level [16].

Identification of an elevated Lp(a) level should prompt more aggressive treatment of modifiable risk factors. In individuals with elevated Lp(a), those with more optimal cardiovascular health, as measured by Life’s Simple 7 score, have significantly decreased cardiovascular risk. However, it should be noted that residual risk remains, and risk factor modification does not fully attenuate Lp(a)-mediated risk [19]. In particular, LDL-C lowering is one of the most powerful tools at the clinician’s disposal to try to address increased cardiovascular risk. In an analysis of the JUPITER trial which randomized individuals to rosuvastatin versus placebo in the primary prevention setting, risk of the primary endpoint was reduced in those with Lp(a) below and above the median, without evidence for a significant interaction, suggesting that, at the least, statin therapy reduces cardiovascular risk among individuals with higher Lp(a) levels [20]. In a large meta-analysis of statin trials, ASCVD risk was evaluated by Lp(a) level and on-treatment LDL-C level. The highest risk was noted when both LDL-C and Lp(a) were high. For any given level of LDL-C however, the risk was greater when Lp(a) was elevated. In particular, even with the lowest achieved levels of LDL-C, there was still a 38 % increased risk when Lp(a) was elevated, demonstrating that LDL-C reduction reduces risk in individuals with elevated Lp(a), but cannot fully offset Lp(a)-mediated risk [14].

Multiple studies have evaluated the effect of Proprotein convertase subtilisin/kexin type 9 inhibitors (PCSK9i) on Lp(a) levels. In a secondary analysis of the FOURIER trial, evolocumab reduced Lp(a) by a median of 26.9 % which appeared to translate into clinical benefit as greater Lp(a) reduction was associated with a greater reduction in cardiovascular risk. Additionally, those with higher Lp(a) levels had a greater absolute risk reduction with evolocumab compared to those with lower levels [21]. A similar secondary analysis of the ODYSSEY OUTCOMES trial demonstrated a greater benefit with the PCSK9i alirocumab with higher Lp(a) levels [22]. In another analysis of ODYSSEY OUTCOMES, among those with LDL-*C* > 70 mg/dL, there was an incremental benefit to PCSK9i only in those with Lp(a) greater than the median [23]. Taken together, these studies suggest greater benefit to PCSK9i in individuals with higher Lp(a) levels. However, while there is potential utility of PCSK9i in individuals with elevated Lp(a), it should be noted that these were secondary analyses and the original trials were not designed to address this issue and thus this is not a class I recommendation.

Another area of increasing interest has been the use of aspirin for primary prevention in individuals with elevated Lp(a) or specific *LPA* genetic polymorphisms. There is a rationale for a particular benefit to aspirin therapy in those with elevated Lp(a) as Lp(a) is associated with increased atherothrombotic risk, possibly due, in part, to pro-platelet effects which may be impacted by aspirin therapy [24]. In an analysis of the Women’s Health Study, the rs3798220 single nucleotide polymorphism (SNP) of the *LPA* gene identified individuals with high Lp(a) levels and increased CVD risk who also experienced a significant reduction in risk with aspirin therapy [25]. Similar findings were demonstrated in an analysis of the ASPREE trial, which also demonstrated a potential net benefit to aspirin therapy with regards to the balance of CVD event reduction and bleeding risk [26]. In an analysis of the Multi-Ethnic Study of Atherosclerosis (MESA), a similar reduction in coronary heart disease events was observed with regular aspirin use in individuals with Lp(a) >50 mg/dL [27]. Finally, a study utilizing data from the National Health and Nutrition Examination Survey (NHANES) observed a reduction in ASCVD mortality with aspirin use in individuals with elevated Lp(a) [28]. These findings require further validation, but, as of now, aspirin may be a reasonable option for individuals with elevated Lp(a) who have not had prior events and are not at increased bleeding risk. Along the same lines, there is evidence for potential benefit to prolonged dual antiplatelet therapy in individuals with elevated Lp(a) in the secondary prevention setting [29,30].

Lipoprotein apheresis is the only FDA approved therapy for elevated Lp(a), currently indicated for individuals with clinically diagnosed with familial hypercholesterolemia, coronary artery disease of peripheral arterial disease, and Lp(a) ≥60 mg/dL. Lipoprotein apheresis results in an acute decline in Lp(a) of approximately 70–80 %, with a time-averaged decreased of 20–30 %. This decrease in Lp(a) appears to translate into a significant reduction in clinical events, though in primarily nonrandomized studies [31].

Identification of an elevated Lp(a) should trigger cascade screening which is an effective method for further identifying individuals with elevated Lp(a). The number needed to screen for first-degree relatives is approximately 2, and approximately 3 for second degree relatives [32]. Furthermore, identification of individuals with elevated Lp(a) supports further needed research and development related to Lp(a), including potential novel therapies.

## 3 Novel therapies

While there are strategies to address the increased risk associated with elevated Lp(a) today, it is clear that residual risk remains, even with LDL-C lowering [14]. Moreover, mendelian randomization studies have suggested that a large reduction in Lp(a) is needed to achieve significant benefit. In one study, a reduction in Lp(a) of 66 mg/dL was estimated to be needed to achieve the same benefit as a 39 mg/dL reduction in LDL-C (approximately 22 % relative risk reduction over 5 years) [33]. As such, there are several novel, targeted therapies for lowering Lp(a) in various stages of development which are eagerly awaited (Table 1).

**Table 1:** Status of Novel Lp(a)-Lowering Therapies in Clinical Trials.

Therapy	Mechanism	Lp(a)-lowering	Current Stage	Population	Anticipated completion
Pelacarsen	ASO	∼80 % in phase 2	Phase 3 Lp(a)HORIZON trial for CVD outcomes(35) Phase 2 Lp(a)FRONTIERS CAVS trial for aortic stenosis(NCT05646381)	CVD secondary prevention Calcific aortic valve stenosis	2026 2030
Olpasiran	siRNA	∼100 % in phase 2	Phase 3 OCEAN(a)-Outcomes trial (NCT05581303)	CVD secondary prevention	2026
Lepodisiran	siRNA	∼94 % in phase 2	Phase 3 ACCLAIM-Lp(a) trial (NCT06292013)	CVD secondary prevention and high risk primary prevention	2029
Zerlasiran	siRNA	∼96 % in phase 2	TBD	TBD	TBD
Muvalaplin	Small molecule	∼86 % in phase 2	TBD	TBD	TBD

ASO = antisense oligonucleotide, siRNA = small interfering RNA.

The therapy which is furthest along in development is pelacarsen, an anti-sense oligonucleotide (ASO) which inhibits the production of apolipoprotein(a) in hepatocytes by targeting *LPA* messenger RNA (mRNA). In a phase 2 trial of pelacarsen, reduction in Lp(a) levels of up to 80 % was observed [34]. An ongoing phase 3 trial, the Lp(a)HORIZON trial, will evaluate whether potent Lp(a) lowering with pelacarsen translates into a reduction in cardiovascular events in a secondary prevention population [35]. The trial is expected to complete in early 2026. Pelacarsen is also being evaluated in the Lp(a)FRONTIERS CAVS trial, which aims to evaluate whether Lp(a) lowering can slow the progression of calcific aortic valve stenosis (NCT05646381).

Olpasiran is a small interfering RNA (siRNA) which also targets apolipoprotein(a) mRNA to reduce Lp(a) synthesis, resulting in placebo adjusted reduction in Lp(a) levels of >100 % [36]. Interestingly, in participants from the OCEAN(a)-DOSE trial, there was a sustained reduction in Lp(a) of 40–50 % approximately 1 year after stopping therapy [37]. The ongoing OCEAN(a)-Outcomes trial is expected to complete at the end of 2026 (NCT05581303).

Lepodisiran is a long-acting siRNA, resulted in potent and sustained lowering of Lp(a) after just a single dose, of up to 97 % initially and up to 94 % at close to 1 year, in a small dose-ascending trial [38]. In a phase 2 trial, lepodisiran resulted in sustained Lp(a) lowering up to 94 % at 6 months [39]. The phase 3 ACCLAIM-Lp(a) trial is currently ongoing with expected completion in 2029 (NCT06292013). Of note, this trial also includes a high-risk primary prevention arm. Zerlasiran is another siRNA that resulted in an up to 96 % reduction in Lp(a) at 36 weeks in a phase 2 trial [40]. Plans for a phase 3 trial are pending. Finally, muvalaplin is the first oral small molecule inhibitor of Lp(a) which works by preventing disulfide bond formation between apolipoprotein(a) and apolipoproteinB100. In a phase 1 study, Lp(a) levels were lowered up to 65 % [41]. In the phase 2 study, Lp(a) was lowered by up to 86 %, and apolipoprotein(a) was lowered up to 70 % [42].

## 4 Conclusions

Though Lp(a) is well recognized as a genetic, causal risk factor for ASCVD, it remains underappreciated and undertested in clinical practice. There are several arguments in favor of testing today (Central Illustration) including the association with multiple cardiovascular diseases, the high yield of testing to identify affected individuals and their family members, the relative simplicity and stability of a single test, and its utility in clinical management. In terms of management, Lp(a) testing can enhance personalized risk assessment, guide aggressive risk factor modification, particularly LDL-C lowering and the use of PCSK9i, and identify individuals who may benefit from preventive aspirin therapy. Finally, while there are strategies for addressing Lp(a)-mediated risk today, there is hope for potent targeted therapies for Lp(a)-lowering in the very near future.

## Funding

Dr. Bhatia is supported by 10.13039/100000002National Institutes of Health, Grant 1K08HL166962 and UC San Diego BEACON Cente.

## Disclosures

Dr. Bhatia - consultant / advisor for Kaneka, Novartis, NewAmsterdam, Arrowhead and Abbott.

## CRediT authorship contribution statement

**Harpreet S. Bhatia:** Writing – original draft, Data curation, Conceptualization.

## Declaration of competing interest

Harpreet S. Bhatia reports financial support was provided by National Institutes of Health. Harpreet S. Bhatia reports a relationship with Kaneka that includes: consulting or advisory. Harpreet S. Bhatia reports a relationship with Novartis that includes: consulting or advisory. Harpreet S. Bhatia reports a relationship with NewAmsterdam Pharma Corporation that includes: consulting or advisory. Harpreet S. Bhatia reports a relationship with Arrowhead Pharmaceuticals Inc that includes: consulting or advisory. Harpreet S. Bhatia reports a relationship with Abbott that includes: consulting or advisory. If there are other authors, they declare that they have no known competing financial interests or personal relationships that could have appeared to influence the work reported in this paper.
